# Paroxysmal Atrial Fibrillation in Cryptogenic Stroke Patients With Major-Vessel Occlusion

**DOI:** 10.3389/fneur.2020.580572

**Published:** 2020-11-12

**Authors:** Ryosuke Doijiri, Hiroshi Yamagami, Masafumi Morimoto, Tomonori Iwata, Tetsuya Hashimoto, Kazutaka Sonoda, Hidekazu Yamazaki, Junpei Koge, Naoto Kimura, Kenichi Todo

**Affiliations:** ^1^Department of Neurology, Iwate Prefectural Central Hospital, Morioka, Japan; ^2^Department of Stroke Neurology, National Hospital Organization Osaka National Hospital, Osaka, Japan; ^3^Department of Cerebrovascular Medicine, National Cerebral and Cardiovascular Center, Suita, Japan; ^4^Department of Neurosurgery, Yokohama Shintoshi Hospital, Yokohama, Japan; ^5^Department of Neurology, Tokai University, Isehara, Japan; ^6^Department of Neurology, Saiseikai Fukuoka General Hospital, Fukuoka, Japan; ^7^Department of Neurosurgery, Iwate Prefectural Central Hospital, Morioka, Japan; ^8^Department of Neurology, Osaka University, Suita, Japan

**Keywords:** cryptogenic stroke, insertable cardiac monitors, major-vessel occlusion, paroxysmal atrial fibrillation, stroke recurrence

## Abstract

**Background and Purpose:** To determine whether acute major-vessel occlusion (MVO) predicts atrial fibrillation (AF) in cryptogenic stroke (CS) patients, we analyzed the association between acute MVO and AF detected by insertable cardiac monitoring (ICM).

**Methods:** We conducted a retrospective, multicenter, observational study of patients with CS who underwent ICM implantation between October 2016 and March 2018. In this analysis, we included follow-up data until June 2018. We analyzed the association of MVO with AF detected by ICM.

**Results:** We included 84 consecutive patients with CS who underwent ICM implantation. The proportion of patients with newly detected AF by ICM was higher in patients with MVO than in those without (41% [12/29] vs. 13% [7/55], *p* < 0.01) within 90 days of ICM implantation. The MVO was associated with AF after adjustment for each clinically relevant factor.

**Conclusions:** MVO was independently associated with AF detection in patients with CS, which suggests that MVO may be a useful predictor of latent AF. It is therefore essential to actively assess latent AF in patients with CS presenting with MVO.

## Introduction

Patients with atrial fibrillation (AF) have a 4.8-fold higher risk of developing a stroke compared to those without (1) and is associated with increased stroke severity (2, 3). Moreover, ischemic stroke patients with AF have higher recurrence rates than those without (3); however, oral anticoagulants substantially reduce the risk of recurrent stroke compared to antiplatelet therapy (4, 5). AF detection in ischemic stroke patients is therefore crucial for determining appropriate antithrombotic therapy for secondary prevention. Even after sufficient diagnostic assessment, the cause of stroke remains undetermined and is diagnosed as cryptogenic stroke (CS) in 9 to 25% of ischemic stroke patients (6).

One-third to one-half of acute large-vessel occlusion is reported to have AF in a randomized control trial and a nation-wide registry (7, 8). Even among the CS patients with MVO, covert paroxysmal AF might be a major cause of stroke. Using a wearable 28-day Holter monitor starting 24–72 h after stroke, large-vessel occlusion in patients with CS was reported to be independently associated with atrial fibrillation detection (9). Recently, long-term recording with insertable cardiac monitoring (ICM) has been shown to be useful for AF detection in CS patients (10). In the current study, we analyzed the association between acute MVO and AF detection by ICM.

## Materials and Methods

### Study Design

The current registry was a retrospective, observational registry that enrolled consecutive patients with ICM implantation for CS through five stroke centers in Japan between October 2016 and March 2018 (11). Inclusion criteria were as follows: (1) cryptogenic stroke, (2) ICM implantation, and (3) follow-up lasting 3 months or more. Written informed consent was waived because the study was retrospective. This study complied with the Ethical Guidelines for Medical and Health Research Involving Human Subjects in Japan and with the Declaration of Helsinki guidelines for investigations involving humans, and all methods were carried out in accordance with the relevant guidelines and regulations for observational studies. The Institutional Review Board of all five institutes approved the current study. The names of institutional review boards of all participating centers are as follows: Institutional Review Boards of Iwate Prefectural Central Hospital, National Cerebral and Cardiovascular Center, Yokohama Shintoshi Hospital, Saiseikai Fukuoka General Hospital, and Osaka university. CS was diagnosed based on the Stop Stroke Study-Trial of Org 10172 in Acute Stroke Treatment (SSS-TOAST) system (12). According to this system, after completing diagnostic tests, the local physicians classified a patient as having a cryptogenic embolism when catheter, computed tomography, or magnetic resonance angiography showed an abrupt vessel cutoff in an otherwise normal-appearing artery, which is a culprit of infarction, when imaging evidence showed complete recanalization of the previously occluded artery, or when multiple acute infarctions were present without abnormality in the relevant vessels. The physicians followed the Japanese proposal for clinical indications for ICM (13). Additionally, transesophageal echocardiography, ultrasonic examination for right-to-left shunt, venous duplex ultrasonography, and special blood tests for thrombosis-hemostasis and other parameters for stroke are recommended. In patients without a 24-h Holter electrocardiogram, the physicians confirmed the cardiac monitoring record for 24 h or more without automated rhythm detection.

### Data Collection

We obtained the following clinical information from the hospital charts: age, sex, CHADS_2_ score (14) after index stroke, congestive heart failure, hypertension, diabetes mellitus, plasma B-type natriuretic peptide (BNP) or serum N-terminal pro-B-type natriuretic peptide (NT-proBNP) levels, premature atrial contraction (PAC) count in 24-h Holter electrocardiogram, left atrial diameter (LAD), MVO, and the number of days from stroke onset to ICM implantation and from ICM implantation to the first AF episode. MVO was defined as an occlusion of the internal carotid artery, middle cerebral artery occlusion (M1, M2, or M3), anterior cerebral artery, vertebral artery, posterior inferior cerebellar artery, basilar artery, or posterior cerebral artery. Given the small sample size, we stratified the continuous variables according to clinically relevant thresholds. Among 66 patients with PACs data, the median and IQR of PAC count were 69 and 20–222, respectively. Frequent PACs were therefore defined as more than 222 PACs per day in this study (15). High BNP or NT-proBNP was defined as >100 pg/mL or >300 pg/mL, respectively (16). Large LAD was defined as a diameter of 45 mm or more measured with transthoracic echocardiogram (17).

### ICM Implantation

ICMs (Reveal LINQ; Medtronic, Minneapolis, MN, USA) were implanted under local anesthesia in the left parasternal position at 45 degrees relative to the sternum above the fourth intercostal space. Devices were programmed to detect AF using the company's unique AF detection algorithm. These algorithms recognize AF by assessing the irregularity of R-R peaks in 2 min intervals. The Medtronic CareLink Network was used to transmit device data remotely. The study physician received an alert if the device detected an AF episode. Detected AF episodes were adjudicated by the study physician. The ICM monitoring data up to June 2018 were used for current analysis.

### Study Outcomes

The primary study outcome was the AF detection rate within 90 days from the ICM implantation, and the secondary outcome was found during total follow-up period. The difference in the AF detection rate and the number of days from ICM implantation to the first AF detection was compared between the MVO and non-MVO groups.

### Statistical Analysis

Clinical parameters were described using the median (interquartile range [IQR]), or number (%), as appropriate for the data type and distribution. Differences between the MVO and non-MVO groups were evaluated using Fisher's exact test for PAF detection and other categorical variables, and the Student *t*-test and Mann-Whitney U test were used for continuous variables. A significance level of 0.05 was used for confidence intervals and other statistical tests. Multivariate logistic regression models were developed to assess the independent association of MVO with AF detection in patients with complete data by adjusting for each of the following clinically relevant factors: age in model 1, CHADS_2_ score after index stroke in model 2, congestive heart failure in model 3, hypertension in model 4, diabetes mellitus in model 5, large LAD in model 6, high BNPor NT-proBNP in model 7, and frequent PAC in model 8. Age (18–21), CHADS2 score (14), congestive heart failure (19), hypertension (19), diabetes mellitus (19), large LAD (17, 18, 21), high BNP or NT-proBNP (16, 22), and frequent PAC (15) were shown to be associated with AF in CS. We compared the number of days from ICM implantation to AF detection between the MVO and non-MVO groups using the log-rank test and Cox proportional-hazards model, adjusting for the above-mentioned factors. All analyses were performed using JMP (version 12.0.1; SAS Institute, Cary, NC, USA).

## Results

### Baseline Characteristics

**Figure 1:**
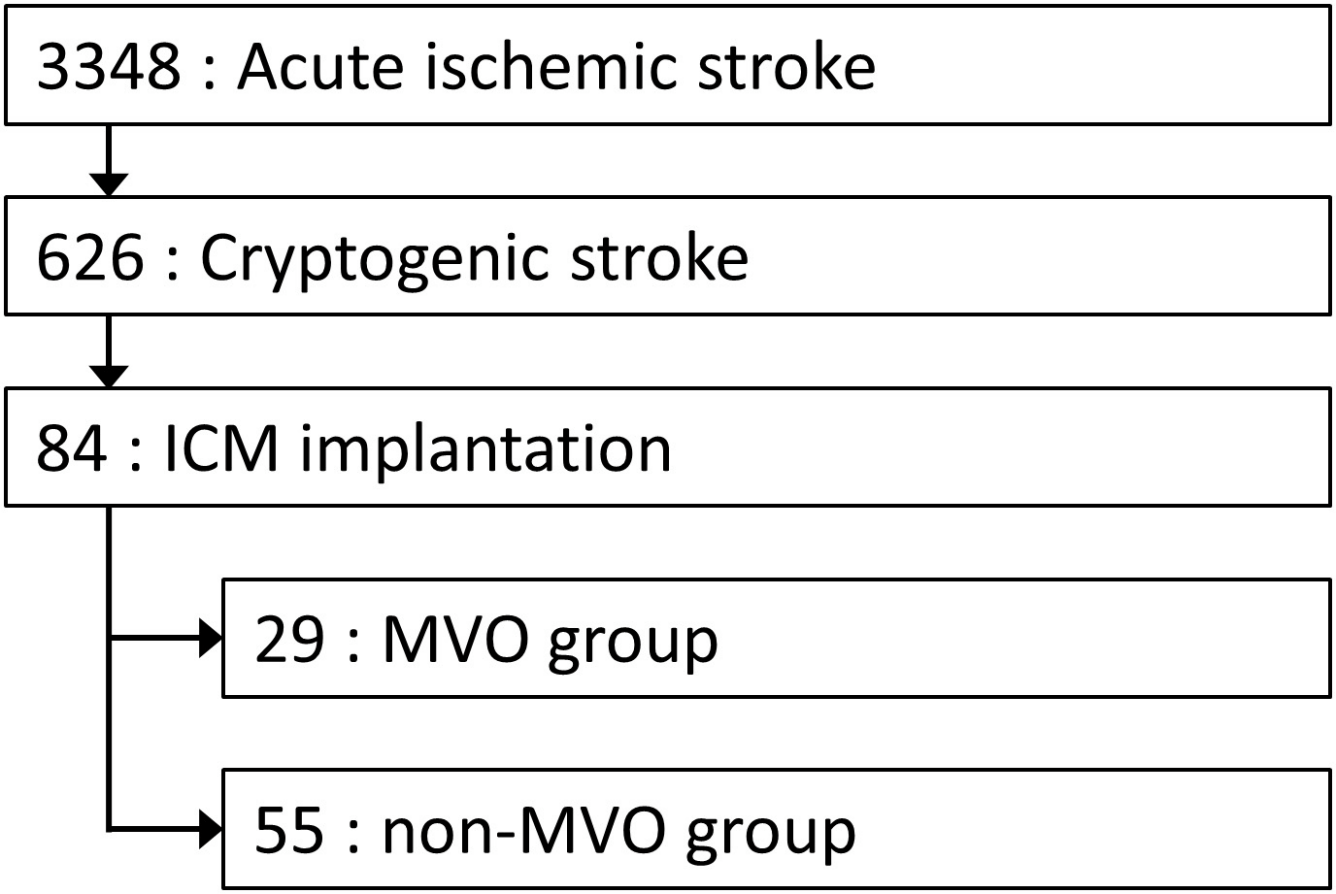
Flowchart of Study Population. Among 3,348 patients with acute ischemic stroke, 84 consecutive patients with insertable ICM implantation were enrolled in the current analysis. ICM, insertable cardiac monitoring; MVO, major vessel occlusion.

During the study period, 3,348 patients with acute ischemic stroke were admitted to the participating stroke centers. Based on the TOAST criteria, 626 patients were diagnosed as CS. Among them, 84 consecutive patients underwent ICM implantation (Figure 1). The median onset-to-implantation period was 21 (IQR 14–39) days. All patients were followed for at least 90 days after the implantation, and the median follow-up period was 218 (IQR 158–345) days. We could not acquire the data of PACs with 24-h Holter electrocardiogram for 17 patients, natriuretic peptide (both BNP and NT-proBNP) for three patients, or LAD for three patients.

### Outcomes

**Figure 2:**
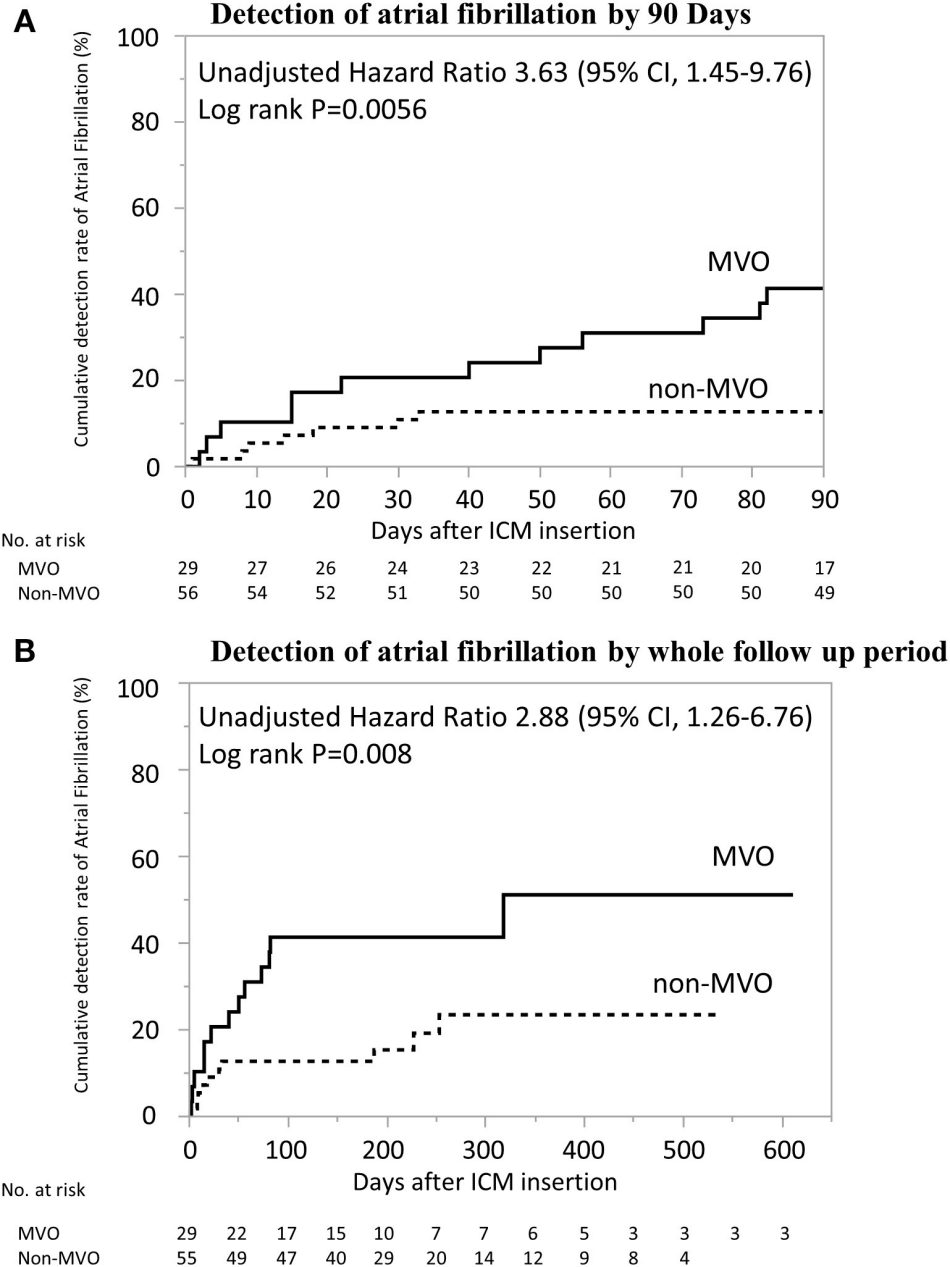
Kaplan–Meier Estimates from ICM Implantation to AF Detection. Days from implantation of insertable cardiac monitoring to AF detection was significantly shorter in patients with MVO than in those without (log-rank *p* < 0.01). **(A)** Detection of atrial fibrillation by 90 days. **(B)** Detection of atrial fibrillation by whole follow up period. AF, atrial fibrillation; ICM, insertable cardiac monitoring; MVO, major vessel occlusion.

The AF detection rate was higher in MVO group than non-MVO group within 90 days and during total follow-up period (41% [12/29] vs. 13% [7/55], *p* < 0.01; 45% [13/29] vs. 18% [10/55], *p* < 0.01, Table 1). Multivariate logistic regression models demonstrated that the adjusted odds ratios [95% confidence interval (CI)] for AF detection significantly increased in MVO group than in non-MVO group, in each model adjusting for age, CHADS_2_ score after index stroke, congestive heart failure, hypertension, diabetes mellitus, large LAD, and high BNP or NT-proBNP (all *P* < 0.05, Table 2). However, the association between MVO and AF detection within total follow up period was dependent on frequent PACs. There was no multicollinearity between MVO and frequent PACs (variance inflation factor = 1.008). The days from ICM implantation to AF detection within 90 days were significantly lower in MVO group than non-MVO group, after adjustment for each clinically relevant factor, and AF detection during total follow-up periods was significantly lower in the MVO group than non-MVO group after adjustment for each clinically relevant factor except frequent PACs (Figure 2 and Table 3).

**Table 1:** Background characteristics and proportion of atrial fibrillation detection.

	**Total** **(*N* = 84)**	**MVO group** **(*N* = 29)**	**Non-MVO group** **(*N* = 55)**	***P*-value**
Age, mean (SD), years	68 (11)	67 (9)	68 (11)	0.54
Male sex, No. (%)	64 (76%)	21 (72%)	43 (78%)	0.56
CHADS_2_ score after stroke, median (IQR)	3 (3–4)	3 (3–4)	3 (3–4)	0.55
Congestive heart failure, No. (%)	5 (6%)	2 (7%)	3 (5%)	0.79
Hypertension, No. (%)	58 (69%)	19 (66%)	39 (71%)	0.61
Diabetes mellitus, No. (%)	17 (20%)	6 (21%)	11 (20%)	0.94
Large LAD^a^, No. (%) (*n* = 81)	12 (15%)	4 (14%)	8 (15%)	0.92
High BNP or NT-proBNP^b^, No. (%) (*n* = 82)	17 (21%)	7 (24%)	10 (19%)	0.57
Frequent PACs^c^, No. (%) (*n* = 66)	16 (24%)	7 (29%)	9 (21%)	0.48
Endovascular thrombectomy, No (%)	17 (20%)	17 (60%)	–	–
Onset-to-implantation period^d^, median (IQR), days	21 (14–39)	18 (14–32)	23 (13-41)	0.73
Whole follow-up period^e^, median (IQR), days	218 (158–345)	199 (156–353)	221 (157–332)	0.77
AF detection through ICM				
Within 90 days from ICM implantation, No (%)	19 (23%)	12 (41%)	7 (13%)	<0.01
During total follow-up period, No (%)	23 (27%)	13 (45%)	10 (18%)	<0.01

*AF, atrial fibrillation; BNP, B-type natriuretic peptide; ICM, insertable cardiac monitoring; IQR, interquartile rage; LAD, left atrial diameter; MVO, major vessel occlusion; NT-proBNP, N-terminal pro-B-type natriuretic peptide; and PAC, premature atrial contraction*.

*Differences between the MVO and non-MVO groups were evaluated using Fisher's exact test for PAF detection and other categorical variables and the Student t-test and Mann-Whitney U test for continuous variables*.

a *Large LAD was defined as 45 mm or more*.

b *High BNP or NT-proBNP was defined as >100 pg/mL or >300 pg/mL, respectively*.

c *Frequent PAC was defined as the highest quartile of the patients enrolled in the current analysis, i.e., more than 222 beats a day*.

d *Onset-to-implantation days were defined as days from index stroke onset to ICM implantation*.

e *Whole follow-up period was defined as days from ICM implantation to the end of follow-up, i.e., until June 30, 2018*.

**Table 2:** Odds ratios for atrial fibrillation detection.

**Model**	**Predictor variables**	**Adjusted odds ratio for AF detection within 90 days from ICM implantation (95% CI)**	***P*-value**	**Adjusted odds ratio for AF detection during total follow-up period (95% CI)**	***P*-value**
1 (*N* = 84)	Age, per 1-year increase	1.02 (0.97–1.07)	0.50	1.02 (0.97–1.07)	0.43
	MVO	5.05 (1.68–15.17)	<0.01	3.82 (1.38–10.57)	<0.01
2 (*N* = 84)	CHADS_2_ score, per 1-point	0.84 (0.47–1.49)	0.63	0.98 (0.57–1.66)	0.95
	MVO	4.81 (1.62–14.26)	<0.01	3.65 (1.33–9.95)	0.01
3 (*N* = 84)	Congestive heart failure	0.75 (0.07–8.02)	0.81	0.57 (0.03–4.57)	0.64
	MVO	4.87 (1.64–14.41)	<0.01	3.69 (1.36–10.37)	<0.01
4 (*N* = 84)	Hypertension	1.54 (0.46–5.18)	0.49	2.18 (0.67–7.12)	0.19
	MVO	5.00 (1.68–14.94)	<0.01	3.93 (1.40–10.95)	<0.01
5 (*N* = 84)	Diabetes mellitus	2.49 (0.71–8.73)	0.15	1.65 (0.50–5.44)	0.41
	MVO	5.04 (1.67–15.25)	<0.01	3.69 (1.35–10.11)	0.01
6 (*N* = 81)	Large LAD^a^	2.17 (0.52–9.00)	0.28	1.48 (0.38–5.81)	0.57
	MVO	4.39 (1.44–13.36)	<0.01	3.25 (1.17–9.02)	0.02
7 (N = 81)	High BNP or NT-proBNP^b^	0.90 (0.24–3.43)	0.88	0.65 (0.18–2.40)	0.52
	MVO	4.66 (1.57–13.86)	<0.01	3.59 (1.30–9.90)	0.01
8 (*N* = 66)	Frequent PAC^c^	2.59 (0.71–9.45)	0.15	3.42 (1.02–11.54)	0.04
	MVO	3.48 (1.03–11.75)	0.04	2.52 (0.81–7.84)	0.11

*AF, atrial fibrillation; BNP, B-type natriuretic peptide; ICM, insertable cardiac monitoring; IQR, interquartile rage; LAD, left atrial diameter; MVO, major vessel occlusion; NT-proBNP, N-terminal pro-B-type natriuretic peptide; and PAC, premature atrial contraction*.

a *Large LAD was defined as 45 mm or more*.

b *High BNP or NT-proBNP was defined as >100 pg/mL or >300 pg/mL, respectively*.

c *Frequent PAC was defined as the highest quartile of the patients enrolled in the current analysis, i.e., more than 222 beats a day*.

**Table 3:** Hazard ratios for atrial fibrillation detection.

**Model**	**Predictor variables**	**Adjusted hazard ratio for AF detection within 90 Days from ICM implantation (95% CI)**	***P*-value**	**Adjusted hazard ratio for AF detection during total follow-up period (95% CI)**	***P*-value**
1 (*N* = 84)	Age, per 1-year increase	1.02 (0.97–1.06)	0.47	1.02 (0.98–1.06)	0.39
	MVO	3.78 (1.50–10.25)	<0.01	3.00 (1.31–7.08)	<0.01
2 (*N* = 84)	CHADS2 score, per 1-point	0.87 (0.53–1.39)	0.58	0.93 (0.58–1.43)	0.75
	MVO	3.59 (1.44–9.67)	<0.01	2.87 (1.26–6.74)	0.01
3 (*N* = 84)	Congestive heart failure	0.88 (0.05–4.24)	0.90	0.71 (0.04–3.41)	0.73
	MVO	3.63 (1.45–9.77)	<0.01	2.88 (1.26–6.77)	0.01
4 (*N* = 84)	Hypertension	1.34 (0.51–4.14)	0.57	1.60 (0.64–4.85)	0.36
	MVO	3.67 (1.48–9.89)	<0.01	2.92 (1.28–6.85)	0.01
5 (*N* = 84)	Diabetes mellitus	2.26 (0.79–5.73)	0.10	1.58 (0.57–3.83)	0.34
	MVO	3.72 (1.49–10.00)	<0.01	2.93 (1.29–6.90)	0.01
6 (*N* = 81)	Large LAD^a^	1.87 (0.53–5.24)	0.27	1.46 (0.42–3.95)	0.51
	MVO	3.37 (1.32–9.19)	0.01	2.70 (1.16–6.42)	0.02
7 (*N* = 81)	High BNP or NT-proBNP^b^	0.87 (0.25–2.43)	0.81	0.65 (0.19–1.75)	0.42
	MVO	3.51 (1.41–9.74)	<0.01	2.83 (1.24–6.67)	0.01
8 (*N* = 66)	Frequent PAC^c^	2.11 (0.70–5.89)	0.17	2.60 (0.99–6.54)	0.05
	MVO	2.62 (0.94–7.89)	0.06	1.96 (0.78–5.00)	0.15

*AF, atrial fibrillation; BNP, B-type natriuretic peptide; ICM, insertable cardiac monitoring; IQR, interquartile rage; LAD, left atrial diameter; MVO, major vessel occlusion; NT-proBNP, N-terminal pro-B-type natriuretic peptide; and PAC, premature atrial contraction*.

a *Large LAD was defined as 45 mm or more*.

b *High BNP or NT-proBNP was defined as >100 pg/mL or >300 pg/mL, respectively*.

c *Frequent PAC was defined as the highest quartile of the patients enrolled in the current analysis, i.e., more than 222 beats a day*.

## Discussion

Our results revealed that AF was more frequently detected through ICM in CS patients with MVO than those without MVO. To the best of our knowledge, this is the first report to demonstrate the association between MVO and higher rate of AF detection using ICM in patients with CS. This association was independent of age, CHADS2 score after index stroke, congestive heart failure, hypertension, diabetes mellitus, BNP or NT-proBNP levels, and LAD but dependent on frequent PACs. However, there was no multicollinearity between MVO and frequent PACs. An MVO may therefore be a useful independent predictor of latent AF in CS. Recently, MVO in patients with CS was reported to be independently associated with AF detection through wearable Holter monitoring within a month of stroke onset (9), which was consistent with our results. However, the patients in our cohort underwent ICM implantation 21 (IQR 14–39) days after stroke onset. It is important to combine wearable monitoring in acute phase and ICM in chronic phase after stroke.

A study assessing the thrombus histology in CS with endovascular thrombectomy revealed that CS and cardioembolic stroke showed a strong overlap in histopathologic characteristics, and both were different from arterio-embolic stroke (23). These findings suggest that a cardioembolic cause of stroke may be predominant among CS patients with acute MVO. Although the histological findings were obtained only from patients with thrombectomy, this may be the same in patients with and without thrombectomy because a large registry of data showed that the distribution of risk factors and stroke subtypes were almost the same between patients with and without endovascular thrombectomy (8). Sufficient examination and assessment for latent AF, including long-term monitoring with ICM, should thus be performed in CS patients with MVO.

Several factors are thought to be predictors of latent AF in CS: increased age (18–21), hypertension (19), diabetes mellitus (19), heart failure (19), higher CHADS_2_ score (14), higher National Institute of Health Stroke Scale score (24), cortical or cerebellar infarcts (25), higher BNP or NT-proBNP levels (16, 22), frequent PACs (15) and atrial run on Holter electrocardiogram (26), and larger LAD on echocardiography (17, 21). Some classes of drugs, including angiotensin II receptor blockers, are suitable candidates for AF prevention (27). From these predictors, we selected age, CHADS_2_ score, BNP or NT-proBNP levels, and LAD as adjusting variables. Even though we did not explore all the potential risk factors for AF, MVO was independently associated with AF. Due to small sample size, we did not develop multivariate analyses by adjusting for all clinically relevant factors simultaneously but developed multivariate analyses separately by adjusting for each factor.

In the Cryptogenic stroke and Underlying AF (CRYSTAL AF) trial, the median time from randomization to detection of AF was 41 (IQR 14-84) days during the 6-month follow-up period and 84 (IQR 18-265) during the 12-month follow-up period (10). Based on these results, our study defined primary outcome as within 90 days from the ICM implantation. In the present study, the detection of AF within 90 days was observed in 12 among 13 cases during the total follow-up period. It suggests that MVO related AF could be detected relatively early after the index CS.

In our stroke centers, although 626 patients were diagnosed as CS according to SSS-TOAST system, only 84 patients underwent ICM implantation. One of the reasons was that the study period was just after the Japanese authority approval in September 2016. In our cohort, the numbers of the patients who underwent ICM implantation in every quarter of the year were 2, 7, 9, 9, 28, and 29, from the fourth quarter of 2016 to the first quarter of 2018. Additionally, Japanese proposals for clinical indications for ICM implantation are stricter compared to international proposals (12). After sufficient workup examination, we thus did not implant ICM in patients with possible symptoms of stroke, including moderate to severe atherosclerosis on aortic arch, right-to-left shunt, abnormal blood tests on thrombosis-hemostasis, cervico-cerebral artery dissection, active cancer, and two or more simultaneous causes. No ICM was implanted in patients without sufficient workup evaluation. Third, given the cost and invasiveness, we avoided implanting ICM in patients with older age or severe disability. These are also the reasons why the detection rate of AF was high compared with previous reports.

This study has several limitations. First, the sample size was relatively small. The small sample size did not allow us to develop multivariate analyses by adjusting for all clinically relevant factors simultaneously. However, despite a small sample size, we identified clearly a strong association between MVO and AF. Second, we did not explore all the potential risk factors for AF and MVO. There may consequently be potential confounding factors. Third, this study was a retrospective and observational study. Prospective studies are required to validate the relationship between MVO and AF. Fourth, among 626 CS patients, only 84 patients underwent ICM implantation and included in the current analysis, leading to potential selection bias. Lastly, there may be some differences in diagnostic workup between patients with and without MVO, which may also cause potential selection bias.

## Conclusion

AF was more frequently detected through ICM in CS patients with MVO than those without MVO. This association was independent of previously known factors associated with AF, which suggests that MVO may be a useful predictor of latent AF. Our data indicated that it is essential to investigate latent AF using long-term recording with ICM in patients with CS presenting with MVO.

## Data Availability Statement

The raw data supporting the conclusions of this article will be made available by the authors, without undue reservation.

## Ethics Statement

The studies involving human participants were reviewed and approved by the Institutional Review Board of all 5 institutes. The name of institutional review boards of all participating centers are as follows: Institutional Review Boards of Iwate Prefectural Central Hospital, National Cerebral and Cardiovascular Center, Yokohama Shintoshi Hospital, Saiseikai Fukuoka General Hospital, and Osaka University. Written informed consent from the (patients/ participants OR patients/participants legal guardian/next of kin) was not required to participate in this study in accordance with the national legislation and the institutional requirements.

## Author Contributions

RD and HY were responsible for the organization and coordination of the study. KT was the principal investigator and responsible for the data analysis. MM, TI, TH, and NK developed the study design. All authors contributed to the writing of the manuscript and approved the final version. All members contributed to the management or administration of the study.

## Conflict of Interest

RD, HY, MM, TI, KS, HY, and KT disclose lecture fees from Medtronic. The remaining authors declare that the research was conducted in the absence of any commercial or financial relationships that could be construed as a potential conflict of interest.

## References

[1] Wolf PA Abbott RD Kannel WB. Atrial fibrillation as an independent risk factor for stroke: the Framingham *study*. Stroke. (1991) 22:983–8. 10.1161/01.STR.22.8.983 1866765

[2] Lin HJ Wolf PA Kelly-Hayes M Beiser AS Kase CS Benjamin EJ. Stroke severity in atrial fibrillation. The Framingham study. Stroke. (1996) 27:1760176–4. 10.1161/01.STR.27.10.1760 8841325

[3] Kimura K Minematsu K Yamaguchi T. Atrial fibrillation as a predictive factor for severe stroke and early death in 15,831 patients with acute ischaemic stroke. J. Neurol. Neurosurg. Psychiatry. (2005) 76:679–83. 10.1136/jnnp.2004.048827 15834026 PMC1739612

[4] EAFT (European Atrial Fibrillation Trial) Study Group Secondary prevention in non-rheumatic atrial fibrillation after transient ischaemic attack or minor stroke. Lancet. (1993) 342:1255–62. 10.1016/0140-6736(93)92358-Z 7901582

[5] Connolly SJ Eikelboom J Joyner C Diener HC Hart R Golitsyn S. Apixaban in patients with atrial fibrillation. N. Engl. J. Med. (2011) 364:806–17. 10.1056/NEJMoa1007432 21309657

[6] Hart RG Catanese L Perera KS Ntaios G Connolly SJ. Embolic stroke of undetermined source: a systematic review and clinical update. Stroke. (2017) 48:867–72. 10.1161/STROKEAHA.116.016414 28265016

[7] Goyal M Menon BK van Zwam WH Dippel DW Mitchell PJ Demchuk AM. Endovascular thrombectomy after large-vessel ischaemic stroke: a meta-analysis of individual patient data from five randomised trials. Lancet. (2016) 387:1723–31. 10.1016/S0140-6736(16)00163-X 26898852

[8] Yoshimura S Sakai N Uchida K Yamagami H Ezura M Okada Y. Endovascular therapy in ischemic stroke with acute large-vessel occlusion: recovery by endovascular salvage for cerebral ultra-acute embolism Japan registry 2. J. Am. Heart Assoc. (2018) 7:e008796. 10.1136/neurintsurg-2018-SNIS.224 29695384 PMC6015290

[9] Pala J Juega J Francisco-Pascual J Bustamante A Penalba A Pala E. Large vessel occlusion is independently associated with atrial fibrillation detection. Eur. J. Neurol. (2020) 27:1618–24. 10.1111/ene.14281 32347993

[10] Sanna T Diener HC Passman RS Di Lazzaro V Bernstein RA Morillo CA. Cryptogenic stroke and underlying atrial fibrillation. N. Engl. J. Med. (2014) 370:2478–86. 10.1056/NEJMoa1313600 24963567

[11] Iwata T Todo K Yamagami H Morimoto M Hashimoto T Doijiri R. High detection rate of atrial fibrillation with insertable cardiac monitor implantation in patients with cryptogenic stroke diagnosed by magnetic resonance imaging. J. Stroke Cerebrovasc. Dis. (2019) 28:2569–73. 10.1016/j.jstrokecerebrovasdis.2019.05.023 31230824

[12] Hakan Ay Karen LF Singhal A Wade S Smith Gregory Sorensen A. An evidence-based causative classification system for acute ischemic stroke. Ann. Neurol. (2005) 58:688–97. 10.1002/ana.20617 16240340

[13] Toyoda K Okumura K Hashimoto Y Ikeda T Komatsu T Hirano T. Identification of covert atrial fibrillation in cryptogenic ischemic stroke: current clinical practice in Japan. J. Stroke Cerebrovasc. Dis. (2016) 25:1829–37. 10.1016/j.jstrokecerebrovasdis.2016.05.012 27282299

[14] Gage BF Waterman AD Shannon W Boechler M Rich MW Radford MJ Validation of clinical classification schemes for predicting stroke: results from the National Registry of Atrial Fibrillation. JAMA. (2001) 285:2864–70. 10.1001/jama.285.22.2864 11401607

[15] Todo K Iwata T Doijiri R Yamagami H Morimoto M Hashimoto T. Frequent premature atrial contractions in cryptogenic stroke predict atrial fibrillation detection with insertable cardiac monitoring. Cerebrovasc. Dis. (2020) 49:144–50. [Published correction appears in *Cerebrovasc Dis*. (2020) 49:1–7]. 10.1159/000505958 32023609

[16] Ponikowski P Voors AA Anker SD Bueno H Cleland JG Coats AJ ESC guidelines for the diagnosis and treatment of acute and chronic heart failure: the task force for the diagnosis and treatment of acute and chronic heart failure of the European Society of Cardiology (ESC). Developed with the special contribution of the Heart Failure Association (HFA) of the ESC. Eur. Heart J. (2016) 18:2129–2200. 10.1093/eurheartj/ehw128 27206819

[17] Hamatani Y Ogawa H Takabayashi K Yamashita Y Takagi D Esato M. Left atrial enlargement is an independent predictor of stroke and systemic embolism in patients with non-valvular atrial fibrillation. Sci. Rep. (2016) 6:31042. 10.1038/srep31042 27485817 PMC4971566

[18] Cotter PE Martin PJ Ring L Warburton EA Belham M Pugh PJ. Incidence of atrial fibrillation detected by implantable loop recorders in unexplained stroke. Neurology. (2013) 80:1546–50. 10.1212/WNL.0b013e31828f1828 23535493 PMC3662328

[19] Benjamin EJ Levy D Sonya M Vaziri SM D'Agostino RB Belanger AJ. Independent risk factors for atrial fibrillation in a population-based cohort. The Framingham Heart Study. JAMA. (1994) 271:840–44. 10.1001/jama.271.11.840 8114238

[20] Thijs VN Brachmann J Morillo CA Passman RS Sanna T Bernstein RA. Predictors for atrial fibrillation detection after cryptogenic stroke: results from CRYSTAL AF. Neurology. (2016) 86:261–9. 10.1212/WNL.0000000000002282 26683642 PMC4733152

[21] Poli S Diedler J Härtig F Götz N Bauer A Sachse T. Insertable cardiac monitors after cryptogenic stroke–a risk factor based approach to enhance the detection rate for paroxysmal atrial fibrillation. Eur. J. Neurol. (2016) 23:375–81. 10.1111/ene.12843 26470854

[22] Okada Y Shibazaki K Kimura K Iguchi Y Miki T. Brain natriuretic peptide as a predictor of delayed atrial fibrillation after ischaemic stroke and transient ischaemic attack. Eur. J. Neurol. (2010) 17:326–31. 10.1111/j.1468-1331.2009.02813.x 19845751

[23] Boeckh-Behrens T Kleine JF Zimmer C Scheipl F Pelisek J Schirmer L. Thrombus histology suggests cardioembolic cause in cryptogenic stroke. Stroke. (2016) 47:1864–71. 10.1161/STROKEAHA.116.013105 27197854

[24] Fujii S Shibazaki K Kimura K Sakai K Aoki J. A simple score for predicting paroxysmal atrial fibrillation in acute ischemic stroke. J. Neurol. Sci. (2013) 328:83–6. 10.1016/j.jns.2013.02.025 23522527

[25] Favilla CG Ingala E Jara J Fessler E Cucchiara B Messe SR. Predictors of finding occult atrial fibrillation after cryptogenic stroke. Stroke. (2015) 46:1210–15. 10.1161/STROKEAHA.114.007763 25851771

[26] Gürdogan M Kehaya S Korkmaz S Altay S Ökan U Kaya C. The relationship between diffusion-weighted magnetic resonance imaging lesions and 24-hour rhythm holter findings in patients with cryptogenic stroke. Medicina. (2019) 55:38. 10.3390/medicina55020038 30720741 PMC6409892

[27] Maggioni AP Latini R Carson PE Singh SN Barlera S Glazer R. Val-HeFT investigators. Valsartan reduces the incidence of atrial fibrillation in patients with heart failure: results from the Valsartan Heart Failure Trial (Val-HeFT). Am. Heart J. (2005) 149:548–57. 10.1016/j.ahj.2004.09.033 15864246

